# Validation of the Glover–Nilsson Smoking Behavioral Questionnaire (GN-SBQ) to Evaluate Nicotine Dependence in Spanish Clinical Settings

**DOI:** 10.3390/ijerph20021119

**Published:** 2023-01-08

**Authors:** José Luis Carballo, Sara Rodríguez-Espinosa, Clara Sancho-Domingo, Ainhoa Coloma-Carmona

**Affiliations:** Center for Applied Psychology, Miguel Hernández University, Avenida Universidad, s/n, 03202 Elche, Spain

**Keywords:** tobacco, smoking, Glover–Nilsson Smoking Behavioral Questionnaire, tobacco use disorder, reliability, validity

## Abstract

An assessment of the different aspects of tobacco addiction is central to adapting interventions to the profiles and needs of smokers. The Glover–Nilsson Smoking Behavioral Questionnaire (GN-SBQ) is one of the few and most used scales to evaluate the behavioral aspects of tobacco addiction. However, few studies involve the validation of the GN-SBQ in clinical settings. Thus, this study aimed to analyze the psychometric properties of the GN-SBQ in a sample of Spanish smokers. A total of 341 smokers attending clinical services in Spain participated in this cross-sectional study. Measures included the psychological factors related to tobacco addiction, assessed with the GN-SBQ, the physical factors of nicotine addiction, withdrawal symptoms, smoking-related variables, and alcohol use. Data analysis included descriptive statistics, internal consistency coefficients, confirmatory factor analyses, Spearman correlations, and the Kruskal–Wallis test. The GN-SBQ showed adequate reliability (α = 0.76 and ω = 0.76) and a unidimensional structure. GN-SBQ scores also provided evidence of convergent and concurrent validity. GN-SBQ scores significantly correlated with the physical symptoms of addiction, age, number of cigarettes, and withdrawal symptoms. The results of discriminant validity were also adequate, as no correlation was observed between GN-SBQ scores and CO levels or alcohol use. Significant differences were found between all levels of psychological addiction based on the GN-SBQ scores regarding physical nicotine addiction, withdrawal symptoms, and age. Thus, this questionnaire is a reliable and valid instrument to assess the psychological aspects of tobacco addiction in smokers in clinical settings. The short length of the GN-SBQ proves advantageous for its use in time-limited assessments, which are common in public health services.

## 1. Introduction

Tobacco use represents the leading preventable cause of premature mortality, morbidity, and disability worldwide [1]. Despite this, tobacco is ranked as the second most consumed psychoactive substance after alcohol [2,3]. The number of smokers is estimated to reach one billion by 2025 [4], and the prevalence of regular smokers is approximately 20% [1].

Smoking behavior is maintained by a combination of positive and negative reinforcing effects generated by repeated exposure to nicotine and cue-induced urges to smoke [5,6]. Prolonged nicotine use produces neuroadaptations in the brain’s reward system that result in withdrawal symptoms in the absence of nicotine. The avoidance of withdrawal symptoms is one of the key factors in the maintenance of smoking behavior and the development of addiction [5,7,8]. However, continued tobacco use is also related to psychological aspects, such as reactivity towards smoking-related cues (e.g., handling and lighting a cigarette, its taste and smell, smoking after meals, etc.) or relaxation and improved mood after consumption [5,7,8,9]. In fact, lower behavioral skills to cope with triggers and higher smoking-related rituals are associated with less probability of continuous abstinence after smoking cessation interventions [10,11].

Given its importance in the development and maintenance of addictive behaviors, the assessment of the physical and psychological symptoms of addiction is essential. Currently, several measures evaluate nicotine addiction, such as the Fagerström Test for Nicotine Dependence (FTND) [12] or the Nicotine Dependence Syndrome Scale (NDSS) [13]. However, they mostly focus on the assessment of physical symptoms. In contrast, few measures have been developed for the assessment of smoking-related behaviors and the psychological parameters of tobacco addiction, one of which being the Glover–Nilsson Smoking Behavioral Questionnaire (GN-SBQ) [14]. The GN-SBQ is a five-point Likert-type scale consisting of 11 items that depict behavioral patterns such as the rituals and habits associated with smoking, the perceptions or feelings of safety that smoking provides, and the reward value of cigarettes. Glover et al. [14] also provided a classification of smokers according to the different levels of behavioral addiction (mild, moderate, strong, and very strong). The GN-SBQ is a quick and easy-to-apply tool that complements the physical measures of tobacco addiction [15]; therefore, it can be used to adapt treatment to the individual needs of smokers [14].

Although scarce in number, previous psychometric studies have indicated that the GN-SBQ has high internal consistency (Cronbach’s α ≥ 0.80) and temporal stability (*r* ≥ 0.86), which validly measures the psychological aspects of cigarette smoking addiction [15,16,17]. However, no studies analyze its discriminant validity with clinical procedures, such as CO-oximetry, which is commonly employed to detect tobacco use in smoking cessation units (SCUs) and in primary care [18]. Despite the wide use of the GN-SBQ in clinical settings and different populations [19,20,21,22], studies analyzing its psychometric properties in the Spanish clinical population are lacking [23].

In Spain, SCUs and primary care play an important role in promoting and implementing programs for smoking cessation [24]. Thus, the psychometric study of the GN-SBQ seems necessary to assess the utility of the questionnaire when used in clinical settings. This study could be also useful to provide insight into the theoretical and empirical understanding of tobacco addiction. In this sense, previous studies have found that the behavioral patterns of nicotine addiction, measured with the GN-SBQ, correlate with severe physical symptoms, more years of smoking, a higher number of cigarettes used per day, and younger age [16,23,25]. Furthermore, both behavioral and physical symptoms have been revealed as predictors of successful smoking cessation and abstinence, and the use of this questionnaire in clinical settings is recommended for choosing proper treatment [19,23,26,27,28].

For all these reasons, the present study aims to analyze the psychometric properties (reliability and validity based on the internal structure and relationship with other variables) of the GN-SBQ in a sample of Spanish smokers.

## 2. Materials and Methods

### 2.1. Participants

This cross-sectional, descriptive study was conducted on 341 Spanish smokers. This sample size was considered sufficient, as it exceeds the minimum 10:1 ratio of individuals per item required to validate assessment instruments [29]. The participants were recruited from two public hospitals in Alicante Province, using a non-probabilistic convenience sampling between January 2014 and January 2020. Inclusion criteria were as follows: aged 18 years or older, being enrolled in care at the Pneumology Units of the participating hospitals due to respiratory problems (e.g., dyspnea, cough, or expectoration), and reporting smoking cigarettes. Smokers unable to provide self-report data, with significant cognitive impairment, history of other substance use disorder withing 12 months before the assessment, and those with incomplete assessments were excluded.

The participants had a mean age of 56.26 years (SD = 9.88), ranging from 21 to 83 years, and included an equal number of men and women, the latter representing 53.1% (*n* = 181) of the sample (Table 1). According to GN-SBQ scores, 25.5% (n = 87) of the smokers were classified as having mild, 51.3% (*n* = 175) moderate, 20.8% (*n* = 71) strong, and 2.3% (*n* = 8) very strong behavioral symptoms of tobacco addiction.

### 2.2. Measures

#### 2.2.1. Psychological Symptoms of Tobacco Addiction

The Glover–Nilsson Smoking Behavioral Questionnaire (GN-SBQ) [14] is an 11-item instrument scored on a Likert-type scale from 0 (not at all/never) to 4 (extremely so/always) that focuses on the behavioral aspects of tobacco addiction. The total score ranges from 0 to 44, classifying smokers into four levels of addiction: mild (<12), moderate (12–22), strong (23–33), and very strong (>33). This study used the Spanish version of the GN-SBQ translated by Nerín et al. [23]. Validation studies in different countries have demonstrated its high internal consistency (Cronbach’s α ≥ 0.80) and temporal stability (*r* ≥ 0.86) [15,16,17]. Regarding the internal structure of the GN-SBQ, the unidimensionality proposed in an original study after conducting principal component analysis [14] was supported by the exploratory factor analyses (EFA) of the validation studies by Rath et al. [17] and Chen et al. [16]. However, the confirmatory factor analysis (CFA) of Rocha et al. [15] revealed a lack of fit for the one-factor model (RMSEA = 0.096 and CFI = 0.857) and found the two-factor structure to be most suitable for the Portuguese version of the scale, as it explained 47.9% of the variance. The first factor was related to smoking habits and rituals, while the second factor involved the behaviors associated with smoking when unable to light a cigarette.

#### 2.2.2. Variables Related to Psychological Symptoms of Tobacco Addiction

##### Sociodemographic and Smoking-Related Variables

Data on age, sex, marital status, academic level, employment status, age at smoking onset, number of years smoking, and number of cigarettes used per day were collected using an ad hoc assessment tool.

##### Tobacco Addiction

The Fagerström Test for Nicotine Dependence (FTND) [12], in its Spanish version [30], consists of four items with dichotomous response options (yes/no) and two Likert-type items (range: 0 to 3) that assess the severity of the physical symptoms of nicotine addiction. The total score ranges from 0 to 10, classifying smokers into three levels of physical addiction: low (<4), medium (5–6), and high (≥7). Previous psychometric studies found Cronbach’s α values between 0.66 and 0.86 [30,31,32].

The Short Nicotine Dependence Syndrome Scale (NDSS) [13], in its Spanish adaptation [33], is a brief 6-item measure scored on a 5-point Likert-type scale that assesses the physical and behavioral aspects of smoking addiction. The total score ranges from 6 to 30, with higher scores indicating a higher level of addiction. This scale has shown adequate reliability in previous studies, with Cronbach’s α values between 0.76 and 0.86 and a test–retest coefficient of 0.76 [13,33,34,35]. The internal consistency of the scale in this study was also adequate, with a Cronbach’s α value of 0.74.

##### Nicotine Withdrawal Symptoms

The Minnesota Tobacco Withdrawal Scale (MTWS) [36] is a 13-item instrument that collects the different symptoms experienced during the previous week that are characteristic of nicotine withdrawal (e.g., irritability, difficulty concentrating, increased appetite). These items are scored on a 4-point Likert-type scale, with the total score ranging from 0 to 39. In previous studies, the scale has shown adequate reliability, with Cronbach’s α values between 0.77 and 0.91 and test–retest coefficients between 0.59 and 0.88 [37,38,39]. The Cronbach’s α in this study was 0.81.

##### Exhaled Carbon Monoxide

Exhaled carbon monoxide (CO) was measured from CO-oximetry, with higher CO levels indicating a greater number of cigarettes smoked daily and higher levels of physical symptoms of tobacco addiction [40].

##### Alcohol Use

The Alcohol Use Disorders Identification Test (AUDIT) [41] collects data on current alcohol use. Three items scored from 0 to 4 (total score range: 0 to 12) were used, including the frequency of standard drinking units (SDUs), the number of SDUs drunk on a typical drinking day, and the frequency of drinking five or more SDUs on a single occasion. Spanish validation studies in primary care patients and populations with and without alcohol dependence have found good reliability of this tool, with internal consistency higher than 0.86 and test–retest reliability of 0.90 [42,43]. The AUDIT showed an acceptable internal consistency in this study, with a Cronbach’s α of 0.71.

### 2.3. Procedure

Trained psychologists conducted individual, structured interviews during consultation hours at the Pneumology Units of the participating hospitals. At the time of assessment, the participants had not yet started smoking cessation treatment. Participation was voluntary, and the participants received detailed information about the study and signed informed consent. No compensation was provided for participation. All procedures were approved by the Clinical Research Ethics Committee of the General University Hospital of Alicante (reference: PI2019/096).

### 2.4. Data Analysis

Data analysis was performed using IBM SPSS Statistics (v.25) (SPSS: Armonk, NY, USA) and the R program (v.3.6.3) (R Foundation: Vienna, Austria) with the packages lavaan [44] and MBESS [45]. The confidence level was set at 95%.

Descriptive statistics, including means with standard deviations and frequencies with percentages, were used to report participant characteristics. The descriptive analysis of the GN-SBQ included skewness and kurtosis indices for each of the 11 items. The absolute values of skewness less than 1 and kurtosis less than 3 were considered acceptable [46]. The corrected item–total correlations were also obtained to establish the discriminative ability of the items, with values equal to or above 0.30 considered acceptable [47]. Reliability was examined with the internal consistency coefficients Cronbach’s α and McDonald’s ω, indicating acceptable reliability scores above 0.70 [48].

Validation analysis of the GN-SBQ was based on the internal structure of the instrument and its relationship with other variables. Regarding factorial validity, CFAs were performed from a matrix of polychoric correlations given the ordinal nature of the items. A one-factor model [14] and two-factor model (Factor 1: Items 1, 2, 4, 5, 8, and 10; Factor 2: Items 3, 6, 7, 9, and 11) [15] were tested. Model parameters were estimated with the robust unweighted least squares method, as it has shown adequate performance regardless of the number of factors and response categories, item skewness, and sample size [49]. The goodness-of-fit indices considered were the chi-square test with Satorra–Bentler correction ($\chi^{2}{}_{SB}$), the Comparative Fit Index (CFI), the Tucker–Lewis Index (TLI), the Parsimony Normed Fit Index (PNFI), the root-mean-square error of approximation (RMSEA), and the standardized root-mean-square residual (SRMR) were obtained. The $\chi^{2}{}_{SB}$ test with an associated non-significant *p*-value indicates adequate overall model fit. CFI values above 0.90, TLI above 0.80, RMSEA between 0.08 and 0.10, and SRMR below 0.08 indicate an acceptable fit of the data to the model [50,51]. As for the PNFI, higher values denote greater parsimony of the model [52]. Modification Indices (MI) were also calculated to examine the fixed parameters that could maximize model fit, provided they were theoretically plausible [53]. An MI above the critical $\chi^{2}$ value of 3.84 (df = 1) was considered significant for an α value of 0.05 [54]. Standardized factor loadings were also obtained for the GN-SBQ items, considering significant values greater than 0.30 [55].

Evidence of the validity of the GN-SBQ was investigated using Spearman’s correlation coefficients. The convergent validity of the GN-SBQ was analyzed relative to the physical symptoms of tobacco addiction assessed with the FTND and the NDSS. The association of the GN-SBQ with CO values and AUDIT scores was used as an indicator of discriminant validity. The relationship between GN-SBQ scores and other theoretically related constructs, including the number of years smoking, the number of cigarettes used per day, and nicotine withdrawal symptoms, was also considered.

The Kruskal–Wallis test (H) and post hoc pairwise comparisons were conducted to analyze the ability to discriminate smokers according to the behavioral aspects of addiction using the four levels of the GN-SBQ. The effect sizes were reported as partial eta squared (*η_p_*^2^) and values of 0.01, 0.06 and 0.14 were considered small, medium and large effects, respectively. The participants with strong (*n* = 71) and very strong addiction (*n* = 8) were grouped together in the analysis of variance to decrease the likelihood of type II error [56].

The pairwise deletion was applied in the analyses involving variables with missing data at random [57]. These variables were age (*n* = 1), the number of smoking years (*n* = 5), the physical symptoms of nicotine addiction based on the FTND (*n* = 2), CO (*n* = 6), and alcohol use (*n* = 1).

## 3. Results

### 3.1. Descriptive Statistics and Reliability

The descriptive analysis of the GN-SBQ items is shown in Table 2. This analysis revealed skewness in Items 2, 3, 6, 7, and 9. Item 6 also showed a positive excess of kurtosis. Most of the items exhibited a good discriminative ability, with item–total correlations higher than 0.40, and an acceptable discriminative ability, higher than 0.30, was observed for Items 7 and 8. Item 3 (“Do you place something in your mouth to distract you from smoking?”) was the only item that had a discriminative power slightly below the established criterion. However, this item remained included considering that its elimination did not improve the internal consistency of the instrument, and hand-to-mouth behavior is one of the most relevant gestures of smoking [58].

The GN-SBQ showed acceptable reliability, with a Cronbach’s α value of 0.757 (95% $CI_{\alpha }$: 0.716–0.796) and McDonald’s ω of 0.762 (95% $CI_{\omega }$: 0.724–0.800). Moreover, all the items significantly contributed to its reliability, as neither Cronbach’s α nor McDonald’s ω increased after the removal of any of the items (Table 2). In contrast, the reliability of the two factors in the validation study of Rocha et al. [15] was below the acceptable limits. Coefficient α values of 0.656 (95% $CI_{\alpha }$: 0.602–0.706) and ω of 0.662 (95% $CI_{\omega }$: 0.610–0.715) were found for Factor 1, and coefficient α values of 0.581 (95% $CI_{\alpha }$: 0.492–0.660) and ω of 0.580 (95% $CI_{\omega }$: 0.488–0.671) were found for Factor 2.

### 3.2. Factorial Validity

CFAs indicated a similar fit for the one- and two-factor models used in this study (Table 3). In both models, the $\chi^{2}{}_{SB}$ test was significant (*p* < 0.05). However, this overall fit index is sensitive to sample size and favors complex models with a larger number of parameters [59,60]. In contrast, the relative fit indices CFI and TLI were acceptable (>0.90), as were the RMSEAs (<0.10). However, the upper limit of the RMSEA confidence interval was slightly above acceptable values in both models. SRMR values were also slightly above the recommendation of 0.08 for an acceptable fit. For this reason, the fit improvement of both models was analyzed using MI. An MI of 65.820 was obtained in the one-factor model [14] and 60.603 in the two-factor model [15], with correlations between the measurement errors of Items 3 and 9. Given that both items refer to placing unlit cigarettes or other objects in the mouth as an avoidance and distraction strategy, the correlation between these measurement errors was considered plausible at the theoretical and content levels. Therefore, the two models were respecified, obtaining a good fit in both according to the CFI value and an acceptable fit according to the TLI, RMSEA, and SRMR values, as shown in Table 3.

**Figure 1:**
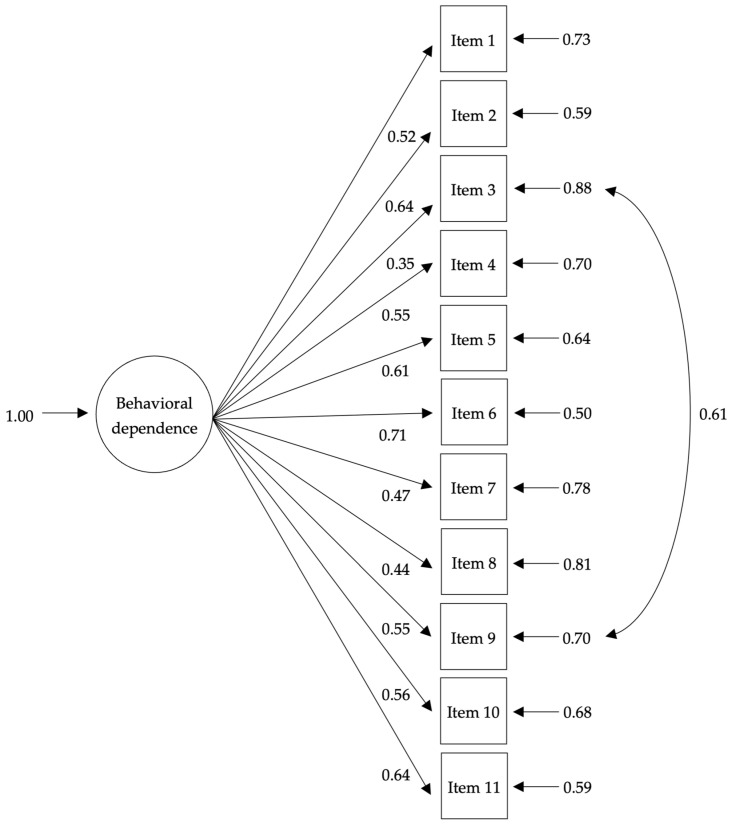
Standardized factor loadings of the one-factor model of the GN-SBQ (N = 341). The one-factor model of the GN-SBQ is illustrated with correlated measurement errors between Items 3 and 9. All items adequately measured the latent factors of smoking behavioral dependence by presenting standardized factor loadings greater than 0.30. Items 2, 5, 6, and 11 showed the strongest relationship with behavioral dependence. These items refer to the manipulation of cigarettes, the difficulty in concentrating in the absence of cigarettes, and the perception of security they provide. Conversely, Items 3, 7, and 8 contributed the least to behavioral dependence and exhibited the greatest level of errors. These items are related to the use of cigarettes by routine or associated with contextual cues, as well as the use of objects to avoid smoking. All factor loadings were significant (*p* < 0.05).

Considering the goodness-of-fit indices, the principle of parsimony [55], and the highest value of the PNFI, the unidimensional model proposed by Glover et al. [14], together with the correlated measurement errors, was finally selected. Although the fit of the model developed by Rocha et al. [15] was similar, the correlation between the two factors was high for the original model (*r* = 0.89, *p* = 0.001) and the model with correlated measurement errors (*r* = 0.95, *p* = 0.001). In addition, the respecified one-factor model showed standardized factor loadings above 0.30 for all GN-SBQ items (Figure 1).

### 3.3. Convergent and Discriminant Validity

Analysis of the GN-SBQ convergent validity relative to other tobacco addiction measures showed moderate and positive correlations with the FTND (*r* = 0.409, *p* = 0.001) and the NDSS (*r* = 0.499, *p* = 0.001). By contrast, the total scores of the GN-SBQ did not correlate with either CO levels (*r* = 0.102, *p* = 0.115) or AUDIT scores (*r* = -0.006, *p* = 0.932), supporting discriminant validity. In addition, FTND and NDSS scores significantly correlated with CO levels (*r* = 0.400, *p* = 0.001 and *r* = 0.177, *p* = 0.004, respectively), which suggests that the GS-SBQ mainly measured the behavioral aspects of addiction (Table 4).

Moreover, higher GN-SBQ scores were related to younger age (*r* = −0.336, *p* = 0.001), a higher number of cigarettes used per day (*r* = 0.143, *p* = 0.008), and more severe withdrawal symptoms (*r* = 0.352, *p* = 0.001), as these measures revealed weak correlations. Conversely, the total score of the GN-SBQ showed a weak correlation with the number of smoking years (*r* = −0.123, *p* = 0.024; Table 4).

### 3.4. Mean Differences between GN-SBQ Levels of Addiction

Significant differences were found between the three levels of the GN-SBQ regarding other nicotine addiction measures (Table 5). Higher scores for measures involving the physical symptoms of addiction were found in the groups with higher scores for behavioral measures, and the effect sizes were large (*η_p_*^2^_FTND_ = 0.152, *η_p_*^2^_NDSS_ = 0.217). More severe withdrawal symptoms were also found in these groups, with a medium effect size (*η_p_*^2^_MTWS_ = 0.132). Smokers with strong behavioral symptoms also showed higher CO levels than those with mild symptoms, but the effect size was small (*η_p_*^2^_CO_ = 0.008). In contrast, groups with higher severity of behavioral addiction were younger, yielding a medium effect size (*η_p_*^2^ = 0.101). On the other hand, although GN-SBQ scores significantly correlated with the number of cigarettes consumed per day, no differences were found between the different severity levels of the psychological symptoms of addiction (*p* = 0.083, *η_p_*^2^ = 0.009). Likewise, no significant differences were found in the number of years smoking (*p* = 0.185, *η_p_*^2^ = 0.004) or AUDIT scores (*p*= 0.735, *η_p_*^2^ = 0.004).

## 4. Discussion

The GN-SBQ is a measure that assesses the behavioral aspects of tobacco addiction and is considered a fundamental measure when adapting smoking treatments to the individual needs of smokers. Although there is a Spanish version of the scale [23], its psychometric properties had not been analyzed in a clinical population thus far. Since most of the tobacco-control strategies in Spain have been promoted in clinical settings [24], the present study aimed to analyze the reliability and validity of the GN-SBQ by analyzing its internal structure and its relationship with other variables in Spanish smokers.

Our results provided evidence for the adequate reliability of the GN-SBQ, with satisfactory Cronbach’s α and McDonald’s ω values (equal to 0.76). Previous validation studies from other countries have reported slightly higher internal consistency of the scale, with Cronbach’s α coefficients between 0.80 and 0.86 [15,16,17].

The unidimensional structure of the GN-SBQ showed an acceptable fit according to the CFI and TLI indices, as observed in the original study by Glover et al. [14] and exploratory studies of Chen et al. [16] and Rath et al. [17]. Considering that the content of Item 3 (i.e., “Do you place something in your mouth to distract you from smoking?”) was similar to that of the Item 9 (i.e., “Do you find yourself placing an unlit cigarette or other objects in your mouth and sucking to get relief from stress, tension, or frustration, etc.?”), residuals of this item pair were allowed to correlate. Fit values of the revised one-factor model improved, confirming the fit of the model to the data. These results were similar to the ones obtained in the two-factor model that was also tested according to Rocha et al.’s proposal [15]. However, the correlation between the two factors was high (*r* = 0.95), and the internal consistency of each factor was below acceptable limits. This result suggests that the GN-SBQ predominantly involves a single factor that evaluates the behavioral aspects of addiction.

Additionally, GN-SBQ items showed adequate and statistically significant standardized factor loadings for the one-factor model (greater than 0.30), demonstrating that each of the items is an adequate indicator of the behavioral features of tobacco addiction.

The moderate and significant associations between scores of the GN-SBQ, the FTND, and NDSS also confirmed convergent and concurrent validity [16,23,61]. Furthermore, there was no significant relationship between GN-SBQ scores and problematic alcohol use and CO levels. Conversely, CO levels were correlated with the FTND and the NDSS, the two measures that evaluate physical features of addiction. These results provide evidence for the discriminant validity of the scale and suggest that the GN-SBQ predominantly assesses behavioral aspects of tobacco addiction, as described in previous works [17].

The validity of the GN-SBQ was also demonstrated based on its relationship with age, the number of cigarettes consumed per day, and withdrawal symptoms. Consistent with other studies, higher behavioral addiction scores were weakly related to younger age [16,23] and a higher number of cigarettes consumed per day [25,61]. By contrast, although smoking dependence has been established as a relevant factor related to smoking maintenance and associated with a greater number of years smoking [16,23,25], this study found that GN-SBQ scores correlated weakly and negatively with the duration of smoking. However, this relationship did not remain significant for GN-SBQ addiction levels. In addition, the association of the FTND and NDSS with the number of years smoking was also not significant. These results could be related to the fact that the smokers in our sample had been using tobacco for an average of 35 years. In this regard, Chen et al. [16] found higher scores on the GN-SBQ in those smokers who had been using tobacco for more than 10 years than those who had not. However, in this study, 95.2% (*n* = 320) of the participants reported more than 10 years of tobacco use.

On the other hand, the GN-SBQ and MTWS scores were positively correlated, indicating that higher scores in behavioral addiction could be related to the experience of more intense abstinence symptoms. Although previous studies have not directly analyzed the relationship between the GN-SBQ and withdrawal symptoms, lower rates of tobacco abstinence have been found in smokers with greater psychological addiction and withdrawal symptoms [23,25].

An important contribution of this study was testing the ability of the GN-SBQ to classify the participants into different severity levels of tobacco addiction. GN-SBQ cut-off scores were able to differentiate smokers according to the degree of severity of the physical symptoms of addiction and withdrawal. We found higher FTND, NDSS, and MTWS scores in the groups with greater psychological addiction according to the GN-SBQ, which supports the cut-off points proposed by Glover et al. [14]. In addition, significant differences were also found between GN-SBQ levels and the age of the participants, as younger smokers reported more severe addiction [16,23].

In contrast, although smokers with greater psychological addiction consumed more cigarettes per day, the differences between the groups were not significant. This finding could indicate that daily tobacco use relates to a greater extent to the physical symptoms of addiction than to its behavioral factors, as it has been suggested in the correlation analyses (*r* = 0.53 vs. *r* = 0.14) of other authors [17,25].

Several limitations should be considered. In this study, we analyzed the ability to classify the levels of behavioral addiction with the GN-SBQ, but only 2.3% (*n* = 8) of the smokers presented severe addiction. Previous studies in the Spanish population have also reported low rates of severe addiction measured with the GN-SBQ [23]. For this reason, future research should include larger samples, allowing comparisons of the four levels of the GN-SBQ with adequate statistical power. On the other hand, the use of convenience sampling to recruit participants could have reduced the representativeness of the sample [62]. However, it is difficult to access a clinical population, and one of the strengths of this study is having a relatively large sample (N = 341). Finally, the use of self-reports could also be associated with the occurrence of certain biases, such as social desirability and recall. These biases were minimized through voluntary participation and by conducting individual face-to-face interviews.

## 5. Conclusions

Psychological and behavioral aspects of tobacco addiction have an important role in smoking quit attempts and cessation [10,11,19]. This study showed that the GN-SBQ is a reliable and valid instrument to assess these dimensions in Spanish smokers attending clinical settings. The scale allows an evaluation of behavioral aspects of tobacco addiction in a short time; therefore, it is suitable for use in time-limited settings, such as public health services.

Strong behavioral cigarette addiction increases the probability of relapse after pharmacological treatments [10]. The GN-SBQ can be used to detect smokers that are more affected by smoking rituals or environmental triggers and, in turn, have a decrease likelihood of maintaining abstinence over time. The assessment of these relevant features can help clinicians to adapt treatments to smokers’ profile and improve adequacy of interventions [26]. The use of this questionnaire can complement measures of the physical symptoms of addiction, which is also key to expand the theoretical knowledge of tobacco addiction [26].

## Figures and Tables

**Table 1:** Sample characteristics.

Variables	Total Sample (N = 341)
Demographics	
Age (years), Mean (SD)	56.26 (9.88)
Women, % (*n*)	53.1 (181)
Marital Status, % (*n*)	
Single	11.7 (40)
Married	62.2 (212)
Divorced	18.2 (62)
Widow	7.9 (27)
Academic degree, % (*n*)	
None	3.8 (13)
Primary education	15.5(53)
Secondary education	52.5 (179)
University education	28.2 (96)
Employees, % (*n*)	40.2 (137)
Tobacco use, Mean (SD)	
Age at onset	17.40 (5.75)
Years smoking	34.97 (12.03)
Daily cigarettes	17.48 (8.31)
GN-SBQ	17.30 (7.51)
FTND	5.46 (2.2)
NDSS	15.83 (5.64)
MTWS	10.55 (6.4)
CO	15.48 (8.24)
AUDIT, Mean (SD)	3.99 (3.08)

SD: standard deviation; GN-SBQ: Glover–Nilsson Smoking Behavioral Questionnaire; FTND: Fagerström Test for Nicotine Dependence; NDSS: Nicotine Dependence Syndrome Scale; MTWS: Minnesota Tobacco Withdrawal Scale; CO: CO-oximetry levels; AUDIT: Alcohol Use Disorders Identification Test.

**Table 2:** Descriptive analysis of GN-SBQ items (N = 341).

GN-SBQ Items	Mean (SD)	Skewness	Kurtosis	Corrected Item—Total Correlation	α if Item Deleted	ω if Item Deleted
1. My cigarette habit is very important to me.	2.35 (1.35)	−0.59	−0.84	0.405	0.739	0.742
2. I handle and manipulate my cigarette as part of the ritual of smoking.	0.50 (1.01)	1.96	2.71	0.405	0.740	0.747
3. Do you place something in your mouth to distract you from smoking?	0.75 (1.15)	1.30	0.50	0.284	0.753	0.759
4. Do you reward yourself with a cigarette after accomplishing a task?	2.39 (1.42)	−0.53	−0.99	0.452	0.732	0.736
5. If you find yourself without cigarettes, will you have difficulties in concentrating before attempting a task?	2.00 (1.52)	−0.07	−1.44	0.476	0.729	0.731
6. If you are not allowed to smoke in certain places, do you then play with your cigarette pack or a cigarette?	0.25 (0.70)	3.37	11.99	0.421	0.743	0.749
7. Do certain environmental cues trigger your smoking, e.g., favorite chair, sofa, room, car, or drinking alcohol?	2.89 (1.21)	−1.10	0.39	0.369	0.743	0.747
8. Do you find yourself lighting up a cigarette routinely (without craving)?	2.54 (1.21)	−0.70	−0.24	0.372	0.743	0.747
9. Do you find yourself placing an unlit cigarette or other objects (pen, toothpick, chewing gum, etc.) in your mouth and sucking to get relief from stress, tension, or frustration, etc.?	0.65 (1.11)	1.62	1.52	0.433	0.736	0.745
10. Does part of your enjoyment of smoking come from the steps (ritual) you take when lighting up?	1.49 (1.47)	0.40	−1.30	0.404	0.739	0.746
11. When you are alone in a restaurant, bus terminal, party, etc., do you feel safe, secure, or more confident if you are holding a cigarette?	1.49 (1.52)	0.42	−1.33	0.485	0.727	0.728

GN-SBQ: Glover–Nilsson Smoking Behavioral Questionnaire.

**Table 3:** Goodness-of-fit indices for the estimated models of the GN-SBQ (N = 341).

Model	$\chi^{2}{}_{SB}$	*gl*	CFI	TLI	PNFI	RMSEA (CI 90%)	SRMR
Original							
One-factor (Glover et al., 2005) [14]	194.386	44	0.925	0.907	0.726	0.099 (0.085, 0.113)	0.092
Two-factors (Rocha et al., 2014) [15] ^†^	188.318	43	0.928	0.908	0.712	0.098 (0.084, 0.113)	0.090
Respecified ^††^							
One-factor model with correlated errors	125.682	43	0.959	0.947	0.735	0.075 (0.060, 0.090)	0.074
Two-factor model with correlated errors ^†^	124.929	42	0.959	0.946	0.718	0.075 (0.060, 0.091)	0.074

$\chi^{2}{}_{SB}$: Satorra–Bentler scaled chi-squared statistic; CFI: Comparative Fix Index; TLI: Tucker–Lewis Index; PNFI: Parsimony Normed Fit Index; RMSEA: root-mean-square error of approximation; SRMR: standardized root-mean-square residual. † Factor 1: Items 1, 2, 4, 5, 8, and10; Factor 2: Items 3, 6, 7, 9, and 11. †† Correlated errors between Items 3 and 9.

**Table 4:** Spearman correlations of GN-SBQ with tobacco-addiction-related variables (N = 241).

Items	1	2	3	4	5	6	7	8	9
1. GN-SBQ									
2. FTND	0.409 **								
3. NDSS	0.499 **	0.0406 **							
4. AUDIT	−0.006	−0.008	−0.025						
5. CO	0.102	0.400 **	0.157 **	−0.029					
6. Age	−0.336 **	-0.245 **	−0.152 *	−0.101	−0.186 **				
7. Years of smoking	−0.123 *	−0.073	−0.051	−0.076	−0.097	0.636 **			
8. Daily cigarettes	0.143 **	0.532 **	0.154 **	0.077	0.475 **	−0.152 *	−0.038		
9. MTWS	0.352 **	0.213 **	0.405 **	−0.104	0.026	−0.120 *	−0.043	0.024	

GN-SBQ: Glover–Nilsson Smoking Behavioral Questionnaire; FTND: Fagerström Test for Nicotine Dependence; NDSS: Nicotine Dependence Syndrome Scale; AUDIT: Alcohol Use Disorders Identification Test; MTWS: Minnesota Tobacco Withdrawal Scale; CO: CO-oximetry levels. * *p* < 0.05; ** *p* < 0.01.

**Table 5:** Mean differences between the three GN-SBQ levels of behavioral addiction (N = 341).

Variables	Mild (*n* = 87) Mean (SD)	Moderate (*n* = 175) Mean (SD)	Strong (*n* = 79) Mean (SD)	H (*p*)	*η_p_* ^2^
FTND	4.32 (2.00) ^a^	5.43 (2.12) ^b^	6.82 (1.84) ^c^	53.37 (0.001)	0.152
NDSS	12.15 (4.49) ^a^	15.86 (5.03) ^b^	19.81 (5.37) ^c^	75.28 (0.001)	0.217
MTWS	7.86 (5.23) ^a^	10.12 (6.05) ^b^	14.44 (6.59) ^c^	46.46 (0.001)	0.132
Age	61.17 (8.88) ^a^	55.60 (9.74) ^b^	52.39 (9.19) ^c^	36.15 (0.001)	0.101
Daily cigarettes	16.43 (8.23) ^a^	17.02 (8.16) ^a^	18.86 (8.98) ^a^	4.99 (0.083)	0.009
Years of smoking	36.23 (13.12) ^a^	35.02 (11.77) ^a^	33.50 (11.37) ^a^	3.38 (0.185)	0.004
AUDIT	3.36 (2.78) ^a^	3.22 (2.81) ^a^	3.23 (2.80) ^a^	0.62 (0.735)	0.004
CO	14.43 (7.09) ^a^	15.28 (8.73) ^ab^	17.01 (8.17) ^b^	4.81 (0.090)	0.008

GN-SBQ: Glover–Nilsson Smoking Behavioral Questionnaire; FTND: Fagerström Test for Nicotine Dependence; NDSS: Nicotine Dependence Syndrome Scale; AUDIT: Alcohol Use Disorders Identification Test; MTWS: Minnesota Tobacco Withdrawal Scale; CO: Co-oximetry levels; H: Kruskal–Wallis test; *η_p_*^2^: partial eta squared. ^a,b,c^ Superscripts indicate significant pairwise differences between GN-SBQ levels.

## Data Availability

The data are not publicly available due to ethical reasons.
